# End-to-End Ultrasonic Hand Gesture Recognition

**DOI:** 10.3390/s24092740

**Published:** 2024-04-25

**Authors:** Elfi Fertl, Do Dinh Tan Nguyen, Martin Krueger, Georg Stettinger, Rubén Padial-Allué, Encarnación Castillo, Manuel P. Cuéllar

**Affiliations:** 1 Infineon Technologies AG, 85579 Neubiberg, Germany; elfi.fertl@infineon.com (E.F.); dodinhtan.nguyen@infineon.com (D.D.T.N.); martin.krueger@infineon.com (M.K.); georg.stettinger@infineon.com (G.S.); 2 Department of Electronics and Computer Technology, University of Granada, 18071 Granada, Spain; rubenpadial@ugr.es; 3 Department of Computer Science and Artificial Intelligence, University of Granada, 18071 Granada, Spain; manupc@ugr.es

**Keywords:** MEMS ultrasonic transducer, pre-processing, Fourier transform, machine learning, HMI

## Abstract

As the number of electronic gadgets in our daily lives is increasing and most of them require some kind of human interaction, this demands innovative, convenient input methods. There are limitations to state-of-the-art (SotA) ultrasound-based hand gesture recognition (HGR) systems in terms of robustness and accuracy. This research presents a novel machine learning (ML)-based end-to-end solution for hand gesture recognition with low-cost micro-electromechanical (MEMS) system ultrasonic transducers. In contrast to prior methods, our ML model processes the raw echo samples directly instead of using pre-processed data. Consequently, the processing flow presented in this work leaves it to the ML model to extract the important information from the echo data. The success of this approach is demonstrated as follows. Four MEMS ultrasonic transducers are placed in three different geometrical arrangements. For each arrangement, different types of ML models are optimized and benchmarked on datasets acquired with the presented custom hardware (HW): convolutional neural networks (CNNs), gated recurrent units (GRUs), long short-term memory (LSTM), vision transformer (ViT), and cross-attention multi-scale vision transformer (CrossViT). The three last-mentioned ML models reached more than 88% accuracy. The most important innovation described in this research paper is that we were able to demonstrate that little pre-processing is necessary to obtain high accuracy in ultrasonic HGR for several arrangements of cost-effective and low-power MEMS ultrasonic transducer arrays. Even the computationally intensive Fourier transform can be omitted. The presented approach is further compared to HGR systems using other sensor types such as vision, WiFi, radar, and state-of-the-art ultrasound-based HGR systems. Direct processing of the sensor signals by a compact model makes ultrasonic hand gesture recognition a true low-cost and power-efficient input method.

## 1. Introduction

**Figure 1:**
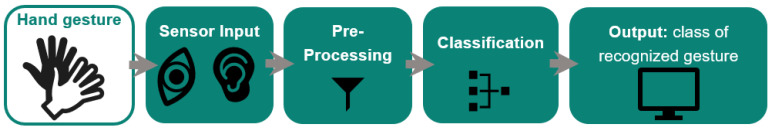
Sensor-agnostic HGR processing flow.

Devices that automate our everyday lives, as in smart cities reported about in [1], and advancements in augmented and virtual reality, as described in [2,3], create a need for new, convenient input methods. HGR is a particularly intuitive form of human–computer interaction (HCI) because people naturally use their hands to communicate with others, as paper [4] states. Additionally, these technologies should be lean regarding cost and power consumption. There are HGR systems based on other sensors such as vision [5,6,7,8,9,10,11], radar [12,13,14], and WiFi [15,16,17,18,19,20,21,22]. Ultrasound-based HGR systems such as those surveyed in [12] or presented in [23] require the user to wear a device to record the hand gestures. Other systems use beamforming, like in [24,25]. The researchers in the latter one additionally use more and bigger ultrasonic transducers. The authors of [26,27] are unable to recognize movements horizontal to the ultrasound sensor array. Other previous ultrasound-based HGR systems use the Fourier transform [28,29,30,31,32,33]. The presented system is hands-free, works solely in the time domain, and can recognize swipe gestures. The visible parts of an HGR system are the hand movement itself, the sensors that record the movement, and a processor. Invisible to the user, processing usually consists of two steps: pre-processing and classification. The aim of pre-processing is to prepare the raw input data for the actual classification, which follows in a second step. Afterwards, the result is typically displayed to the user. Figure 1 presents this HGR processing flow, which is independent of the type of sensors used. Several sensor types have already been considered for HGR to improve user experiences for HCI. In the following, vision-, radar-, WiFi-, and ultrasonic-based systems are compared based on current research. This comparison can be done regarding pre-processing, classification, accuracy, number of gestures, and type of gestures. Pre-processing ranges from channel state information (CSI) and different types of Doppler maps to raw image or radar data. All Doppler maps are based on the Fourier transform. CNN classification is used on data from all sensor types. There are extremely accurate systems for data from every sensor type. But the level of detail in the classified gestures varies substantially as the sets of customized hand gestures being detected differ significantly in granularity, complexity, and number of gestures. Vision-based systems can recognize words through lip reading, and radar-based ones can recognize letters from sign language, while WiFi- and ultrasound-based systems require more coarse gestures. The versatile algorithms for pre-processing and processing available for vision- and radar-based systems show that these technologies are the most researched ones to date. Upon comparing sensor types, it becomes clear that ultrasound cannot achieve the same accuracy or number of gestures as vision- and radar-based systems but requires the least power and can be realized with low-cost HW. Difficulties of ultrasound-based HGR are summarized in Table 1. Table 2 emphasizes the comparison of different sensor types, and Table 3 points out differences among ultrasound-based systems. To the authors’ knowledge, this is the first implementation of ultrasound-based hand gesture recognition using raw echo data and, in fact, the first implementation in the time domain only. Our solution incorporates swipes, in contrast to most previous ultrasound-based HGR systems. All classification is done on the basis of the data from only two or three MEMS ultrasonic transducers used as receivers. Furthermore, even the sender is a MEMS ultrasonic transducer, which has very low output pressure. For this reason, our received signals come with very low SNRs. Nevertheless, we classify our data using gate-based, convolution-based, and transformer-based DL architectures. Each of the architectures requires a specific input data shape, which we come up with on the basis of our domain knowledge from our ultrasound data, along with iterations of tests to discover which input shape produces the best results. Finally, we do manual model tuning for all presented classification algorithms.

The remainder of the manuscript is structured as follows: Section 2 presents an overview of the SotA in HGR through research work on the topic and covers several different sensor types and explains the used pre-processing and classification methodologies. Specific challenges of ultrasound-based HGR are described in Section 2.3. Furthermore, this paper is placed in the SotA. Section 3 characterizes the used HW and explains the datasets. After that, Section 4 explains the classification methodology. Then, a discussion of the findings is given in Section 6. In Section 7, conclusions are made based on the results. Finally, this paper closes with possible future research directions.

## 2. Related Work

This section introduces SotA research on HGR based on different sensors. The systems are divided into non-ultrasound- and ultrasound-sensor-based solutions. Presented non-ultrasound sensors are camera, radar, and WiFi. Table 4 emphasizes the advantages and drawbacks of each of the solutions to facilitate the selection of sensor types for future research.

### 2.1. HGR-Based on Non-Ultrasound Sensor Systems

#### 2.1.1. Sensor-Fusion-Based Systems

The highest accuracy for most fine-grained HGR techniques is achieved through sensor fusion. By combining several sensor types, the information captured with one sensor makes up for the drawbacks of the others while providing the best resolution in various conditions. Examples of such sensor fusion approaches are [34,35]. In the first one, camera and depth information are fused in combination with a skeleton-based approach. In the latter, audio and visual data are combined. While such sensor fusion approaches allow for high accuracy for fine-grained gestures and even lip reading, in this paper, the focus lies on rudimentary gestures that can be recognized with a simple, low-cost, low-power system that can run on an edge device.

#### 2.1.2. Vision-Based Systems

Many vision-based HGR systems are compared in survey paper [5]. Gesture recognition is based on color, appearance, motion, skeleton, depth, a 3D model of a hand, and deep learning. The pre-processing and classification methods compared in Table 2 refer to deep-learning-based classification. The table furthermore gives a comparison of the different approaches and methodologies with respect to the HW used, input image resolution, type of segmentation, feature extraction, classification, results, and possible application areas. Most of the approaches mentioned in [5] are capable of real-time processing. Vision-based systems can employ many already available image processing tools, but they raise privacy concerns and are sensitive to low light and fog.

#### 2.1.3. Radar-Based Systems

Radar-based solutions for HGR are analyzed in [12], which takes into account pulsed and continuous wave radar. Processing algorithms range from rules-based to deep learning, as stated in Table 2. Radar is a promising technology since it preserves privacy and is cost- and power-efficient. Data preparation before classification is similar to ultrasound data pre-processing. The investigated pre-processing approaches include using the amplitude over time, evaluating the amplitude at specific distances, looking at changes in distance, evaluating the change in the Doppler frequency or the change in frequency over time directly, as well as simultaneous use of range and change of Doppler shift at a specific range. Resolution is dependent on the carrier frequency and the propagation speed. Radar operates at higher frequencies than ultrasound but also has a higher propagation speed. Therefore, the resolution depends on which radar and ultrasonic sensor systems are compared. However, radar attenuation in air is significantly lower, especially for higher carrier frequencies. Therefore, ultrasound is only recommended in close proximity when high resolution is required. Radar uses more power than ultrasound and requires HW that is too expensive for certain use cases. Furthermore, ultrasound can be used in harsh environment and is robust to electromagnetic interference.

#### 2.1.4. WiFi-Based Systems

Another technology used for HGR is WiFi. As discussed in paper [15] and stated for comparison in Table 2, the main techniques use the received signal strength indicator (RSSI), channel state information (CSI), frequency modulated carrier wave (FMCW), and Doppler shift. Especially when using FMCW and Doppler shift evaluation algorithms, radar, with its higher frequency bandwidth, is more suitable for HGR. Due to the lower resolution of WiFi compared to radar, it is more appropriate for applications like counting people or fall detection. An advantage of WiFi is that it is readily available in most indoor environments, but it has too low spatial resolution for HGR. Another drawback is that for accurate localization of objects like hands, constant transmission is required. Due to changing usage of WiFi communication in normal use cases, the signal keeps changing and simultaneous use of the WiFi signal for HGR and communication is difficult. Furthermore, changes in the surroundings have a significant effect on WiFi localization and causes a recurring need for retraining. Nevertheless, ref. [17] shows that gesture recognition with high accuracy is possible with an attention-based algorithm on a public dataset.

### 2.2. Related Work on Ultrasound-Based Systems

Ultrasound-based HGR systems offer several potential advantages, including luminance invariance, immunity to electromagnetic interference, high detectability, cost-effectiveness, small form factor, and energy efficiency. The exploration of ultrasound-based HGR traces its origins to the paper [28], which was written in 2012, where the classification of five gestures, including one- and two-handed ones, was based on echo amplitude size and frequency shift. The method proposed in [31] introduces a system that harnesses channel impulse response, which means a pre-defined training sequence of different frequencies is being sent. Sending such a sequence, which is easier to find in the received data, permits more robust and accurate classification. This technology allows for 7 mm resolution and classification of 12 different finger gestures. Later, the research in papers [15,29] showcases approaches to reduce the necessary training data and time. In reference [30], a semi-supervised learning approach is implemented with a focus on incremental training that involves expanding the training set. This makes the model adaptive to variances in the execution of the same movement between different persons. By contrast, reference [31] relies on one-shot learning in combination with a time-dilation-inspired algorithm to make up for those differences while keeping training time low and dataset size at a minimum. Other recent research aims to lower energy and area requirements for HGR. The work [32] presents how generic finite impulse response filter modules can be used to simplify the needed processing structure. Paper [33] compares TOF and spectrogram pre-processing. The survey in [4] describes systems that require the user to wear a device. Such a requirement makes the gadget really unhandy and is not seen as an option in this work. A very recent survey [16] focuses on wireless and acoustic sensing principles and describes the latest ultrasonic hand and finger gesture tracking systems. Among them are technologies based on a mix of extracted features and different transceiver configurations. Two main trends can be distinguished. One relies on increasingly sophisticated classical signal processing methods for pre-processing and another one pursues more robustness and power and chip-area savings through less processing complexity and smaller datasets. The work proposed in [16] already indicates that machine learning (ML) can help with selecting the most relevant features but does not yet propose to skip any feature extraction and leaves extraction of the relevant information to the ML model. The methods proposed in [13,14,36] show that purely ML-based classification of radar and accelerometer data is possible for HGR or human motion recognition. Memory-based algorithms introduced in [6] are provided for automated feature extraction for gesture recognition based on camera and infrared sensor data. An amplitude-based model allows the ML model to automatically learn which features in the raw echo data content are relevant for the given classification task. Table 3 highlights differences between ultrasound-based HGR regarding pre-processing, classification, accuracy, number of gestures, and type of gestures. Current research in ultrasound-based HGR is aimed at increasing the robustness of the systems; lowering the required energy, area, and processing; and lowering training time and the size of the needed dataset. Recently, attempts have been made to replace manual feature selection with automated, ML-based feature selection.

**Figure 2:**
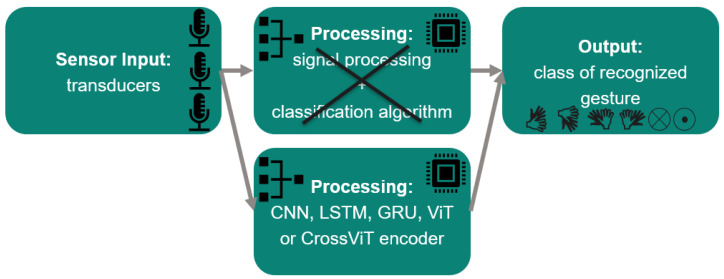
Suggested change in HGR processing flow.

This paper presents a novel approach to HGR based on ultrasound, wherein raw or nearly raw echo samples are employed as input. Almost raw means that minor data processing like a weak low-pass filter may still be applied. However, computationally intensive processing steps, such as the Fourier transform as employed in previous works [5,12,15,29,33], are excluded. Cross-attention multi-scale vision transformer (CrossViT), vision transformer (ViT), original transformer networks, LSTM, gated recurrent unit (GRU), and convolutional neural network (CNN) models were tested on the task of classifying gestures with three different ultrasound receiver arrangements. To the authors’ knowledge, this is the first implementation of ultrasound-based HGR exclusively in the time domain. The proposal is, as depicted in Figure 2, to train CNN, LSTM, GRU, ViT, and CrossViT on ultrasound amplitude values instead of processed data to avoid the Fourier transform in during pre-processing. The papers [13,14] present similar approaches for HGR on radar data.

### 2.3. Challenges of Ultrasound-Based HGR

This section deals with general difficulties of ultrasound-based HGR, while Section 3.2 elaborates in greater detail on the challenges of the specific data gathered with the dedicated HW presented in this work. Ultrasonic sensing for object detection, object localization, distance measurement, imaging, and similar tasks is based on the reflection of ultrasonic waves off of surfaces. An ultrasonic transducer transmits a short pulse towards a possibly reflective object. The reflected wave is detected by the same or different transducer, which converts the audio signal into an electrical one as sampled by means of an analog-to-digital converter. The signal generated in this way is analyzed in different innovative ways explained in this work.

Challenges of ultrasound-based HGR systems can be categorized as those caused by the physical characteristics of ultrasound, those caused by the HW needed for emission and detection of ultrasound waves, and general challenges. All of them are summarized in Table 1.

Among the physical properties that pose challenges for HGR are wind, humidity, and temperature, which all influence the speed of the wave significantly. Furthermore, pulses are not sharply defined, as ultrasound is a mechanical wave generated and received by a membrane with a certain inertia. That means that at the beginning and end of the pulse, there is a time of increasing amplitude after the first actuation of the membrane and a time of ring-down after the last electrical actuation of the membrane. Therefore, the sent and received pulses have a bell shape. Moreover, the surroundings of the system have an influence. Objects in the field of view (FOV) cause unwanted echos and can deflect the wanted signal. Since we are using a low-cost MEMS transducer array, we use only two to three echo streams for gesture classification. Thus, only coarse hand gestures are detectable. The resolution does not allow for finger gesture analysis or the detection of sign language. As we are using transducers instead of dedicated sender and receiver HW, there is more variability in choosing which transducer is used for sending. But this comes with the drawback of lower output pressure and lower sensitivity, which leads to a lower SNR. The lower frequency of ultrasound used in this approach with regard to propagation speed compared to other technologies causes lower resolution. Moreover, low-cost ultrasonic setups have only a few transducers, limiting the amount of available data compared to vision-based systems or high-end medical ultrasonic systems. For the development of an ML-based HGR, no public training data are available. There are indeed lots of HGR datasets for vision-based systems but not for ultrasonic ones. Hence, for this work, our own experimental echo datasets were acquired using a proprietary setup. The processing speed needs to be high to allow for reactions that feel natural for human gestures, which are as short as 0.5 s or even briefer. The maximum sound pressure that can be emitted by the MEMS transducers at the frequency of 23 kHz used in the scope of this work is 71 dBSPL, measured at a distance of 10 cm from the transducer. This is comparable to conversational speech at a distance of 1 m. Cats can hear 20 Hz frequencies at sound pressures as low as 0 dB, and dogs, depending on the race, can hear these at about 20 dB. The attenuation coefficient of ultrasound in air at 20 °C is 750 times higher than in water at 23 kHz. Furthermore the used MEMS transducers emit ultrasound omnidirectionally. Therefore, the sound pressure declines at a rate of $\frac{1}{distance}$. On the other hand, the processing of the ultrasonic echo data for HGR is complex. Several distinct processing steps, as depicted in Figure 1, are usually needed for ultrasound-based HGR. In the first step, ultrasound data are captured by the dedicated HW module. In the second step, the gathered information is transferred to another device used for processing. The processing step is split into a pre-processing part, which is dedicated to performing data transformation tasks and feature extraction. The selected data for which a movement, hand, or gesture was detected is then further passed to the classification module. The module for classification may contain another pre-processing shield to condense the information in the data. Finally, the output of the classification needs to be shown in a final module that displays the result. In our case, the classification entirely consists of an ML algorithm that does end-to-end processing from the raw input to the output classes. This approach is further explained in Section 4, and the specific processing flow is depicted in Figure 2 in the same section. Previous work, by contrast, always involved some kind of pre-processing before classification, such as those described in [5,15,33], which use the Fourier transform, and in [29], which compares Doppler, frequency-modulated continuous wave, channel impulse response, and differential channel impulse response. However in our case, the goal is for the model to autonomously identify the relevant data content for HGR on its own by means of a DL model. The fact that the sensor units regarded in the scope of this work consist of four transducers, of which three act only as receivers, means that only three data channels have to be considered. Consequently, the amount of data that need to be processed in our case is significantly lower than in cases of image processing or ultrasound applications for medical purposes.

Previous work on ultrasound-based HGR is usually based on features like distance calculation or spectrograms. One possible way to do HGR is based on localization of the hand by means of range. Distance calculation is done using a methodology called time-of-flight (TOF). A pulse is emitted from the sending transducer, reflected by the hand in the FOV of the sensor device, and captured by the receiving transducers. TOF is the time calculated between transmission and reception of the ultrasonic pulse. This TOF is proportional to total distance traveled by the pulse. Combining the traveled distance to the different receiver antennas allows estimation of the localization of the hand.

The following relationships can be used for gesture classification. To overcome the challenge that the difference in delay of echos received by different receivers is low, the receivers can be placed with higher distances between each other or in respect to the sender. Furthermore, a smaller distance between the hand and receiver plane causes a higher difference in echo delay with respect to the total delay between the sent pulse and the echo. Movements horizontal to the receiver plane cause higher distance difference shifts than movements perpendicular to the receiver plane. The horizontal location of a hand with respect to the receivers along a line through them can be determined using the sign of the difference of the distances measured by the two receivers. Reference [33] uses TOF data. Up to now, however, distance calculation from ultrasound data from micro-electromechanical system (MEMS) ultrasonic transducers has been imprecise, and previously explained relationships do not reliably appear. Another method for ultrasound-based HGR is spectrogram evaluation. In this case, spectrograms are computed using the Fourier transform to allow for evaluation of changes in frequency over time. Frequency changes caused by the Doppler effect are evaluated in this case. A shift towards a higher frequency compared to the frequency of the sent pulse represents movement towards a receiver, and a lower frequency indicates movement away from the transducer. Even two transducers, one sending and one receiving, are enough to evaluate different gestures, including swipes. The difference between swipes is caused by the fact that the location of the sender and the receiver in relation to the hand are different depending on the direction of the swipe; this therefore leads to a different spectrogram pattern. In spectrogram-based approaches, different movement speeds and hand sizes have an especially high influence on correct classification. Most previous ultrasound-based HGR systems, like [5,12,15,29], use this type of pre-processing.

Using the same ultrasonic transducer for sending the pulse and receiving the echo is difficult, as the membrane that is actuated to send out the pulse has a ring-down time, during which it keeps moving despite not being actuated any more. The ring-down amplitude is larger than the amplitude of the echo; therefore, during this time, the echo cannot be detected. In other terms, if an object is very near to the transducer, no detection is possible. The object and the echo will interfere with the ring-down in some way. The work proposed in [37] deals with those effects and presents an approach on how to reduce them. The pattern repeats itself at multiplications of the lambda distance, and the change in size of the echo pattern is not big enough to determine the distance, at least not in our case. Additionally, lambda is very small, 17.15 mm, so the pattern repeats itself after a very short time. Wind, humidity, and temperature affect the speed of ultrasound. Wind can have an especially detrimental effect on applications using the TOF or spectrogram, as wind comes from one direction and might shift the signal speed in one direction only—but not constantly nor changing directions. Those effects can be reduced when the system is used indoors.

## 3. Hardware and Dataset

**Figure 3:**
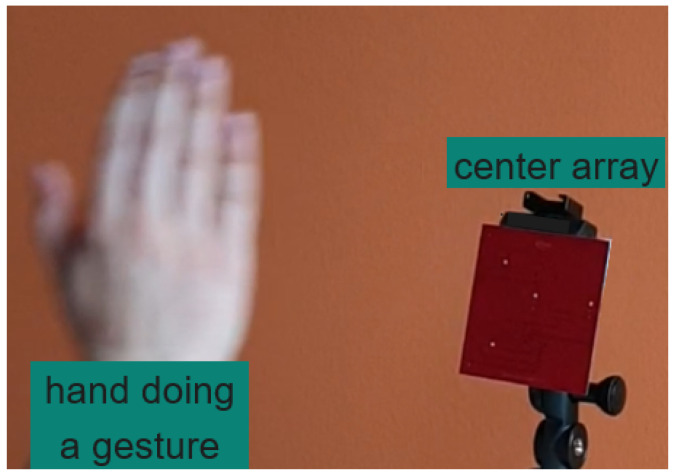
Data acquisition setup.

This section outlines the HW employed for data acquisition and introduces both the acquired data and the dataset. Figure 3 shows one of the transducer shields in relation to a user’s hand during a gesture.

### 3.1. Acquisition Hardware

This section introduces the three transducer arrangements and the processing HW used for data acquisition and explains the parameters used in the acquisition process.

To account for the difficulties explained in Section 2.3 in our work, time windows of 250 pulse trains of raw echo data corresponding to 4 s are evaluated. That is a time window that is a bit longer than a hand gesture takes on average.

Findings based on the classification of the data of the linear shield with either distance or spectrogram pre-processing and a comparison thereof are described in paper [33].

**Figure 4:**
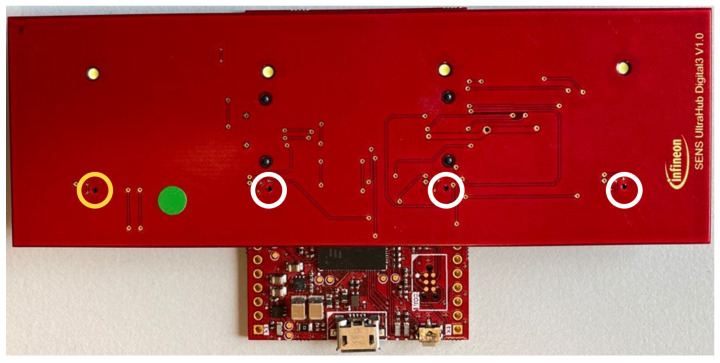
Processing shield with linear array; transducer locations are marked with yellow and white circles; yellow circle—sending transducer and white circle—receiving transducer.

**Figure 5:**
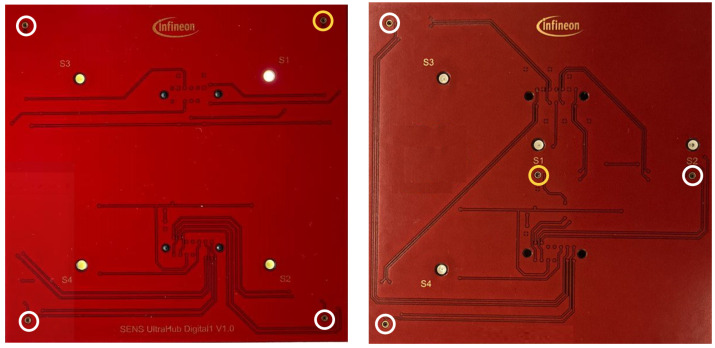
Corner (**left**) and center (**right**) transducer array without processing shield; transducer locations marked with yellow and white circles; yellow circle—sending transducer and white circle—receiving transducer.

The data acquisition HW consists of a sensing shield with four transducers, and it is connected to a processing shield. For this work Infineon digital transducers [38] were used that directly output the received amplitudes via pulse-density modulation (PDM). The processing shield controls transmission and reception of the transducers and has a USB interface through which the received echo signals are sent to, for example, a laptop as a data stream. It leverages a FT900Q microcontroller by Bridgetek, Glasgow, Scotland [39]. Configuration details, like the pulse repetition time, can be stored in an EEPROM on the transducer shield. Further information about the HW setup can be found at [40]. There are three sensing shields with different transducer configurations. In all cases, the transducers are placed at 3 cm distance from each other. Each of them was used to acquire one dataset. Figure 4 shows the linear shield connected to the processing shield. Figure 5 shows a schematic of the corner shield on the left and a figure of the center shield on the right. The active transducer is circled in yellow in Figure 4 and Figure 5. A more detailed description of the HW can be found in paper [27].

### 3.2. Datasets

**Figure 6:**
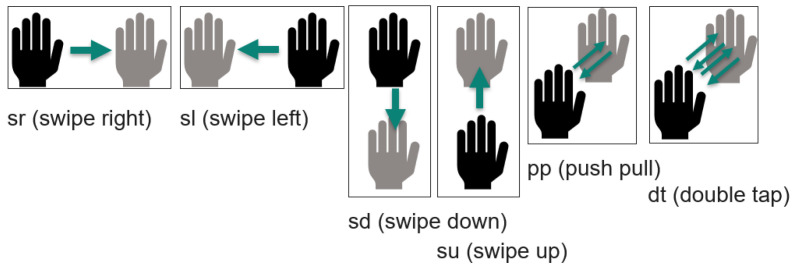
Gestures used in the dataset.

The acquired datasets are called “linear”, “center”, and “corner”, respectively. Each of them is separately assessed in the scope of this paper. Figure 6 shows which gestures are classified. The four gestures swipe right (sr), swipe left (sl), push pull (pp), and double tap (dt) are available for all datasets. Due to the structure of the transducer arrays, swipe up (su) and swipe down (sd) are not part of the linear dataset. Table 5 shows the settings for all cases of data acquisition for this research.

**Figure 7:**
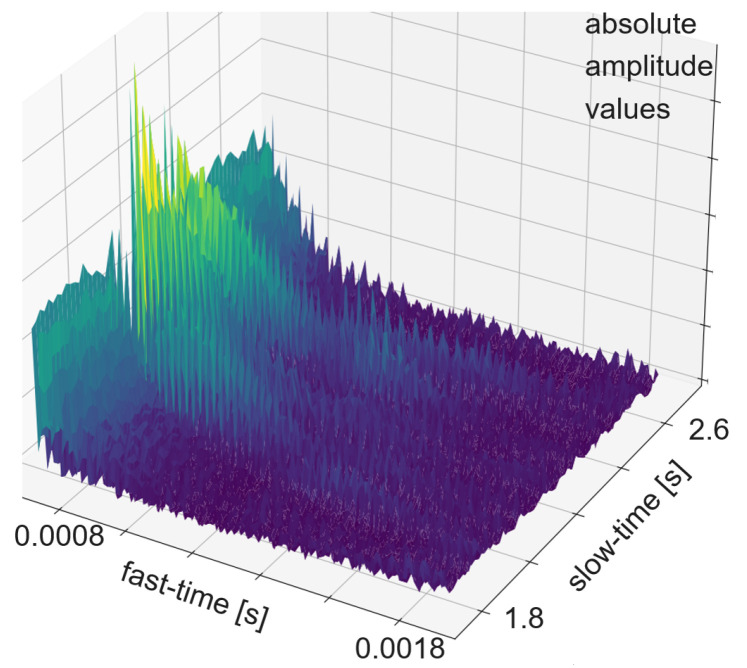
Part of a gesture frame of one channel of a pp gesture. Find an illustration of the pp gesture in Figure 6.

**Figure 8:**
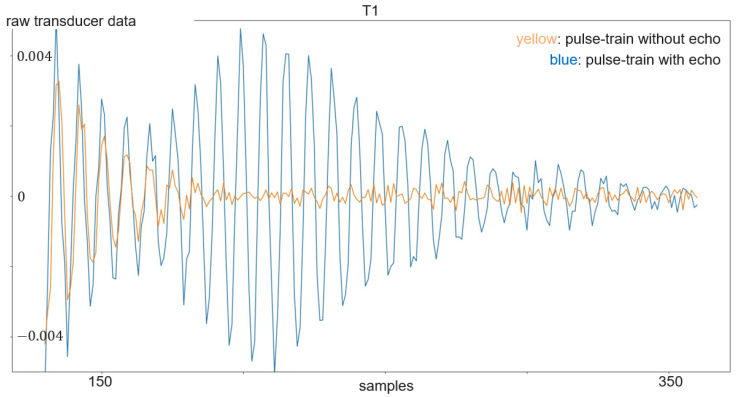
Plot of a pulse train with echo (in blue) compared to a pulse train without echo (in yellow) of transducer 1.

**Figure 9:**
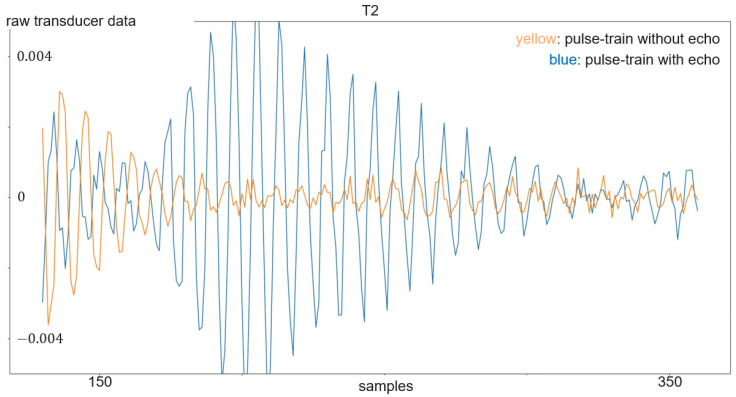
Plot of a pulse train with echo (in blue) compared to a pulse train without echo (in yellow) of transducer 2.

**Figure 10:**
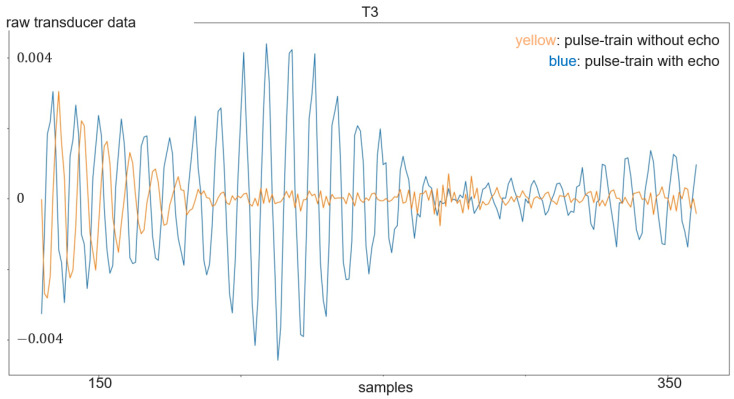
Plot of a pulse train with echo (in blue) compared to a pulse train without echo (in yellow) of transducer 3.

The transmitted signal consists of pulse trains, with each including a sent pulse at the beginning. On the other hand, every frame consists of 250 pulse trains. The sent pulse consists of five wavelengths with a frequency 24 kHz and is emitted every 16 ms from one of the transducers, while all four transducers act as receivers. The sampling frequency is 192 kHz, which is more than sufficient to resolve the 24 kHz of the sent pulse. With each of the three transducer arrangements presented in Section 3.1 and illustrated in Figure 4 and Figure 5, a separate dataset was acquired. First, experiments were carried out with the linear array. Its dataset consists of 899 gesture frames of four different gestures executed by nine test persons. The received data size is 768,000 × 4 with 250 pulse trains of 3072 samples each per transducer. The linear arrangement has three received amplitude values of which only two are used for processing; the center and corner arrangement use three receiving transducers as input for classification. The linear array allows for 2D gestures only, while the center and corner shields permit 3D gestures. Therefore, center and corner datasets have two additional gestures compared to the linear one: su and sd. For the linear case, the classification outputs one of four classes, while the center and corner case outputs one of six classes. Each of the datasets consists of 898 to 900 gesture frames in total executed by six persons. To make the data more diverse, data acquisition was carried out on different days. Figure 7 shows the relevant part of the data of one channel of a gesture frame, and Figure 8, Figure 9 and Figure 10 show the relevant parts of the pulse trains of channels one to three, respectively. The amplitude of T0 is too big to show in the plot as it from the sending transducer. Since the transducers are close to each other, there is a direct path of the sent signal between the sender and all of the receivers. The time differences between the arrivals along the direct paths might be used to determine the distance of the transducers in relation to each other, but in this work, the locations between the transducers are given. Thus, the differences in the direct paths might be used to synchronize timing of the recording. But here, the difficulty is that the echo is not a single peak but a repetition of the sent pulse with a Gaussian bell-shaped envelope. This is caused by the fact that sound waves are mechanical waves that are generated and received by a membrane. In both cases, when the wave is generated as well as when the echo is received, the membrane takes some time to increase its amplitude and, after the end of the excitation time, it takes time to stop oscillating, which spreads the beginning and end of the echo. Consequently, determining the delay between the transmitted pulse and the echo becomes challenging. Figure 8, Figure 9 and Figure 10 show the amplitude values of the receiving transducers at the beginning of a pulse train, after the direct path has subsided. The x-axis corresponds to the number of samples inside one pulse train, starting after the actuation, and the y-axis corresponds to the amplitude value. Up to about sample 175, both signals have about the same amplitude, which corresponds to the end of the actuation by the direct path. At this point, the pulse train without echo, plotted in yellow, and the pulse train with echo, depicted in blue, diverge. The blue signal rises again until about sample 210. This increased amplitude corresponds to an echo reflection at a distance of 0.19m. Those echo signals have different characteristics. Each signal captured by the transducers displays echoes, with each echo characterized by length, amplitude, and temporal position in relation to the transmitted pulse.

Depending on the array configuration, 2D or 3D localization becomes feasible by analyzing the temporal position of the echo in the signals from the different transducers. Unfortunately, the length of the echo varies, and the highest peak of the echo is not always in the same location inside the echo. Since the transducers are very close to each other, the differences between the echo positions are very small, and it is difficult to determine the exact location of the object that is the cause of the reflection. Furthermore, noise may not be homogeneous across different sensors, especially in the time domain. The amplitude of the echo gives further information about the distance of the object to the sender, as ultrasound is attenuated in air. Therefore, a higher distance means a smaller amplitude. Differences in amplitude are too small to be used to determine the differences of the distances. This is caused by the low amplitude emitted. The amplitude is this low as it is difficult to emit high amplitudes with the capacitive (MEMS) ultrasonic transducers used in this work [38]. Stationary objects show a constant echo in the data, while moving objects, like a hand doing a gesture, create a changing echo. As for dynamic HGR, the relevant object is a moving hand; it makes sense to remove static echos in the data. Those static echos are caused by the environment. But since in our case the goal was to use ML on raw data, no removal of static objects was carried out. We relied on the ML system to understand automatically that those static echos, if present, do not have meaning and therefore should not be considered for feature selection and classification. In contrast to a static object, a moving hand causes a change in frequency over time, which is caused by the Doppler effect. That means the echo length is reduced if the hand’s movement is towards the sensor, and it is increased for movements away from the sensor. In the spectrogram, this additionally manifests as a decreased or increased frequency. As described in Section 2.3, an approach for spectrogram-based gesture classification has been described in other papers. The special characteristic of the system presented in this work is that it uses raw amplitude values as input for the ML system instead of features derived using the Fourier transform or distance features generated by correlation. It allows the ML system to learn autonomously which features are relevant for the classification task while avoiding processing intensive pre-processing. This approach is further elaborated in Section 4.

## 4. Classification Methodology

Following the trend of feeding the full given data to an ML system to let the system learn by itself which data content is important and simultaneously avoiding the Fourier transform, no pre-processing was applied to the acquired data in any of the algorithms laid out in this paper. This change in processing flow is depicted in Figure 2 and is the main novelty of the presented work. The ML model types CNN, GRU, LSTM, original transformer, ViT, and CrossViT are trained with our data for the end-to-end HGR task. All of the models are implemented as part of the new processing flow and compared with regard to their usefulness in this context. That means self-attention, gating mechanisms, like in LSTM and GRU, and convolution are used and compared in this work for their usability for classification that includes feature detection for HGR with ultrasound data. Table 6 summarizes parameters of the datasets and classifications carried out in the scope of this work.

Our data can either be regarded as time-series or image data. The CNN structure was used as this is the most widespread and researched model type for image data. GRU and LSTM are specialized RNN models developed for time-series data. The biggest drawback of RNN is the vanishing and exploding gradient problem. LSTM and GRU were developed to counteract this issue. That is because they come with a memory component that allows connections to be found inside the data even if the related information is far apart in terms of time. This is made possible by gating mechanisms. Transformer is a model type that uses attention instead of a gating mechanism for processing sequences. In the scope of this paper, models based on gating mechanisms, self-attention, and convolution were compared with regard to their applicability for the given task. For the attention-based models, warmup and cosine warmup were used to lower the learning rate during the first few steps to help the attention mechanisms slowly adapt to the data. All models were trained with the input of all three channels for 3D classification using the center and corner array and with two-channel input for 2D classification with data from the linear array.

### 4.1. Transformer

In 2014, the attention mechanism was introduced to the world of ML [41], and transformers were introduced in 2017 [42]. Transformers have not only been successfully applied to the task they were originally planned for, natural language processing, but have proven useful for many other tasks as well. The goal of the original transformer, as described in [42], is to reduce sequential computations. All the convolutions and sequential computations in [42] are replaced by an attention mechanism. Multi-head attention especially allows for higher parallelizing and reduced training time. Transformers became known for their high accuracy. ViT, introduced in [43], and CrossViT, described in [44], are very successful adaptations of the original transformer for image data. Our work extends the use of these structures to ultrasound data.

The encoder part of the structure was implemented to verify if transformers improve the accuracy of ultrasound-based HGR. The decoder side is not needed, as ultrasound-based HGR is a classification problem.

In the original version, input images are split into tokens. As our data have sequences of 16 ms corresponding to the pulse repetition time (PRT), this length of time and integer multiplies of it are input tokens in our case. Linear embeddings of these token vectors are input in the transformer model for supervised learning. While the basic transformer is position agnostic, ViT and CrossViT have position embedding. This is crucial in our application because the position of a specific pulse train with respect to previous and later pulse trains determines the direction of, for example, a swipe. Furthermore, the embedding contains an additional classification token that represents the meaning of the whole input sequence.

CrossViT is an adaptation of ViT with the goal of combining good accuracy with low computational complexity. To this end, instead of splitting the input into patches with the same length, in this case, the data are split into two branches: one with short sequences, called "s-branch", and the other with long sequences, called "l-branch". Both branches are processed in parallel. The information from both the long and the short sequences is merged through interaction of each one’s classification token with the token of the other sequence from the other branch. For a visualization of the ViT or CrossViT model, see the respective papers [43,44]. To create the long and short patches for CrossViT, small integer multiplies, up to 18, of the pulse train were used. The length of both patches was determined through trial and error. In our case, the dimension of the embedding vector for ViT was set to 768. The number of stacks for the l-branch for CrossViT was set to three. The s-branch uses one stack with one encoder. Many of the ViT and CrossViT hyperparameters were tested. The combinations that led to the best accuracies on the respective datasets are described in the results in Section 5. In summary, we adapted our models to work on 1D vectors instead of 2D vectors as in the original task for ViT and CrossViT, which are both fairly new architectures that were introduced in 2020 and 2021, respectively.

### 4.2. CNN

**Figure 11:**
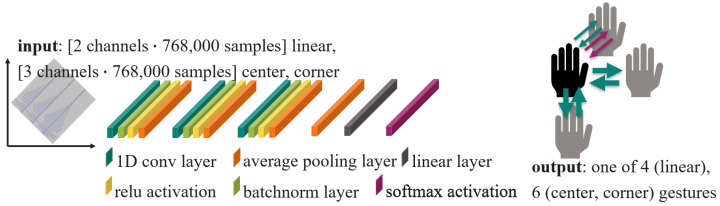
Schema of the CNN model with the best results. When the input is two channels (linear dataset), the output is 1 of 4 possible gestures. When the input is three channels (center and corner dataset), 1 out of 6 gestures is the output.

Given the extensive research in image processing utilizing CNNs, a CNN was selected as one of the model structures for training. The CNN structure implemented in this paper is based on [45]. When CNNs are used for image processing tasks, its convolution layers are 2D and the kernel has a square shape to extract low-level features from the images. Data from two transducers for the linear dataset and three transducers for the center or corner dataset are used for classification. The data recorded by the sending transducer cannot be used, as the ring-down time interferes with the echo, as explained in Section 2.3. In contrast to image processing, in our case the data, are stored in gesture frames of 250 pulse trains with 3072 values from four transducers. Therefore, we adapt the model to 1D convolutions and find the best kernel size for our data to be 80. The implemented CNNs consist of two to four groups of combined sub-layers, consisting of a 1D convolutional layer, a normalization layer, a ReLU function, and either a max pooling layer or an average pooling layer, then a global average pooling layer, a linear layer, and a softmax layer. Figure 11 shows a schema of the CNN model.

### 4.3. GRU and LSTM

LSTM consists of an input, output, and forget gate. The forget gate is meant to assure that valuable information can be retained from much earlier in the sequence while the effect of recent, unnecessary information is diminished. In this way, information far earlier in a sequence that is needed to make sense of a current input can be retained. In our case, this allows us to determine the direction of a swipe gesture.

GRU, like LSTM, uses gates, but it consists of only two gates: a reset gate and an update gate. Thus, it consists of fewer parameters and weights. That means it comes with lower training times and processing requirements and requires less storage space.

Usually in HGR, the spectrograms are used in a feature selection process for feeding the LSTM or GRU with features. In our case, by contrast, we input the raw values of two or three channels with 768,000 data points each that were acquired at a frequency of 192 kHz from the transducers of the linear, center, or corner board and correspond to a gesture frame of 250 pulse trains with 3072 values. Since we omit the spectrogram, we implement two alternative approaches to extract features from the raw amplitude values. Both architectures are learnable during the training of GRU or LSTM to optimize the feature selection process.

This means that for both LSTM and GRU a novel embedding inspired by the one used for ViT is implemented. Its difference is that it comes without the position embedding and the class token embedding. This shape is then transformed using either convolution or a linear transformation. In the first case, the convolution embedding, the input goes through a 1D convolution along the sequence length and has output channels equal to 768, and the kernel size and stride are equal to the patch size. In this way, one dimension of the output tensor is always set to 768, and the other dimension varies depending on patch size. These dimensions correspond to the input size and input length, respectively, of the LSTM/GRU. A transpose function is then applied to ensure that the output tensor conforms to the required shape for LSTM/GRU. In this way, the 1D convolution decides which features are derived from the raw amplitude values. As it is learnable during training, the patch embedding is trained in conjunction with the LSTM or GRU to produce optimal results, which means the final learned embedding’s parameters are varied depending on the specific configurations of the models.

In the second case, for linear embedding, the data first have to be brought into a different shape. The reshaping process ensures that the pulse train repetitions from different receiving transducers stay together. The output of the transformation is a tensor of the shape batch size, the number of pulse train repetitions, the number of channels, and the lengths of pulse train repetitions. The next step consists of a linear transformation framed by layer normalization. Following this process, the input is reorganized so that the information from a small time frame, a small integer number of pulse trains across two to three sensors, is grouped together. These groups, i.e., patches, are flattened. Utilizing layer normalization and a linear layer, this 2D tensor is mapped to the shape expected by the following classification network: LSTM or GRU. In this way, the timing information is retained. One dimension of the output of the transformation is defined to be 768. The other one depends on the patch size, as the data are transformed in chunks of patch size. Depending on the integer value the pulse train is multiplied by, there is a final part of the gesture smaller than a patch. These data are omitted, as it is unlikely that the end of a gesture contains significant information needed for gesture classification. LSTM and GRU are both fed with a 2D data array with a first dimension of 768. The second dimension depends on the patch size. The patch size is an integer multiple of one pulse train of 3072 values, which corresponds to 16 ms.

The implementations of GRU and LSTM used in our experiments have a feature size of 768 features in the input and 2304 features in the hidden state. Different optimization methods and patch sizes and the effect of label smoothing are tested.

## 5. Results

This section first describes general findings on the dataset and the classification performance, followed by a more detailed description of the findings concerning each of the ML models.

The gestures evaluated are illustrated in Figure 6. In each of the datasets, the first few gesture frames per gesture and person contain additional variations compared to the gesture frames in the middle of each of the acquisitions. That is why we manually ensure that the validation datasets contain gesture frames from all phases of the acquisition.

**Figure 12:**
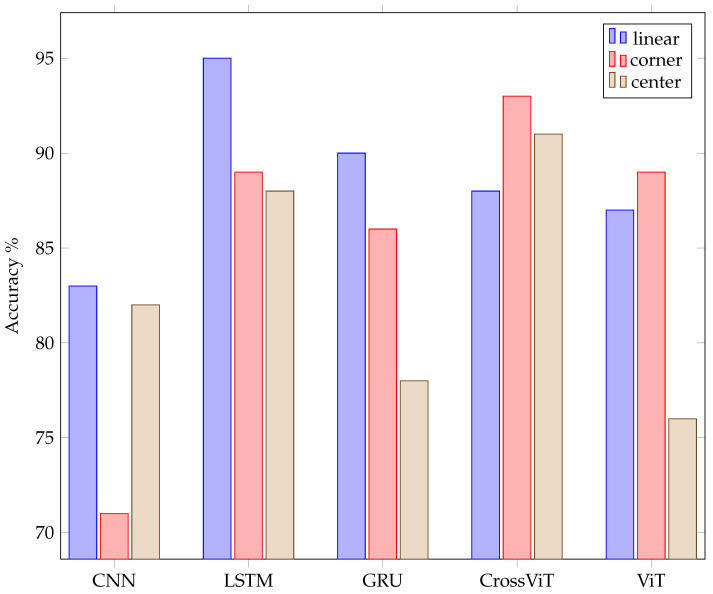
Accuracies per model per dataset.

Table 7 shows the best accuracies achieved independent of the dataset per ML model. It demonstrates that accurate gesture classification is possible using only amplitude values and completely omitting the Fourier transform. This is the first time that deep learning alone was used on ultrasound gesture data. The confusion matrices shown in Table 8, Table 9, Table 10, Table 11 and Table 12 and the summary of accuracies achieved using the different models on the three datasets in Figure 12 give more detailed information about the models’ performance.

In the matrices, columns correspond to model predictions, and rows correspond to the actual classes. The predicted gestures belong to the categories tap or swipe. Tap gestures, dt and pp, consist mainly of a movement perpendicular to the transducer, while for swipe gestures, sd and su, the movement is horizontal to the transducer array. As these groups are considerably different, in tap vs. swipe, we evaluate the classification accuracy of the correct group membership. Accurate classification is calculated as described in Equations (1)–(3).

(1) $$\mathrm{accuracy}\;\mathrm{of}\;\mathrm{taps}=\frac{1}{2}\sum \left(dt,dt\right),\left(dt,pp\right),\left(pp,dt\right),\left(pp,pp\right)$$

(2) $$\mathrm{accuracy}\;\mathrm{of}\;\mathrm{swipes}\;\mathrm{linear}\;\mathrm{dataset}=\frac{1}{2}\sum \left(su,su\right),\left(su,sd\right),\left(sd,su\right),\left(sd,sd\right)$$

(3) $$\begin{array}{c} \mathrm{accuracy}\;\mathrm{of}\;\mathrm{swipes}\;\mathrm{corner}\;\mathrm{and}\;\mathrm{center}\;\mathrm{dataset}\\ =\frac{1}{4}(\sum \left(su,su\right),\left(su,sd\right),\left(su,sr\right),\left(su,sl\right)+\\ \sum (sd,su),(sd,sd),(sd,sr),(sd,sl)+\\ \sum (sr,su),(sr,sd),(sr,sr),(sr,sl)+\\ \sum (sl,su),(sl,sd),(sl,sr),(sl,sl)) \end{array}$$

The average accuracy of only the four gestures pp, dt, su, and sd on the center and corner dataset is not improved compared to the accuracy of all six gestures. By contrast, a comparison of tap versus swipe gestures shows a significantly higher accuracy exceeding 95% in all cases. Furthermore, the accuracy for detecting the angle of the swipe, (sr, sl) vs. (su, sd), calculated according to Equations (2) and (3), exhibits a similarly high accuracy. That means classifying tap gestures versus swipe gestures and classifying perpendicular swipes, like (sd, su) vs. (sl, sr), is easier than determining the direction of swipes and counting the number of taps per gesture frame.

**Figure 13:**
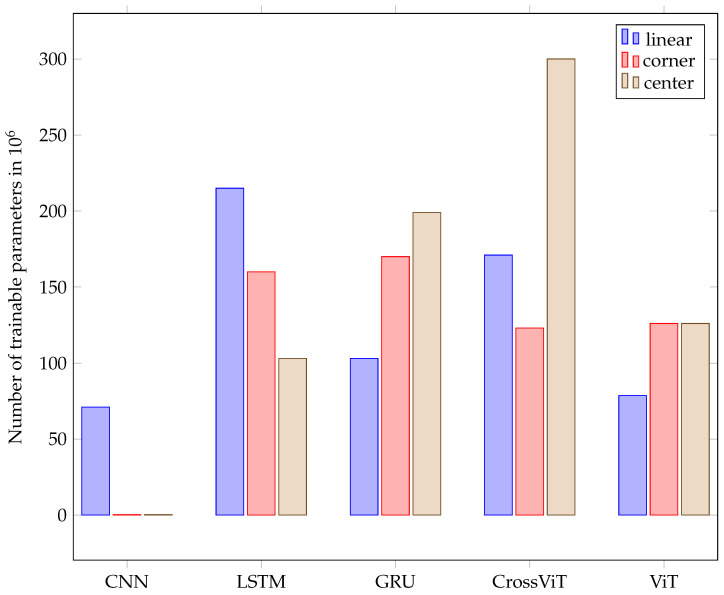
Sizes of best models.

Figure 13 shows the number of parameters with respect to the model and dataset. The number of parameters required for the best CNN model is noticeably smaller than that required for the other models, except for the linear shield. But even for the linear shield, the CNN model required the fewest parameters.

For GRU, CrossViT, and ViT, the highest number of parameters is required by the best models for the center shield. For CNN, center and corner shield both have a similarly small number of required parameters: 283 K. The LSTM model exhibits the lowest parameter requirement, 103 M, for center shield, coupled with a fairly good accuracy rate of 88%. For a comparison of the parameter values of all models, see Figure 13. Despite classifying only four gestures, the model for linear data is the smallest for GRU and ViT. The best models for the corner data showed the least variance.

The best overall models and dataset are, as displayed in the bar plot of Figure 12, LSTM on linear data, with 95% accuracy, followed by CrossViT on corner data, with 93% accuracy, and CrossViT on center data, with 91% accuracy.

**Figure 14:**
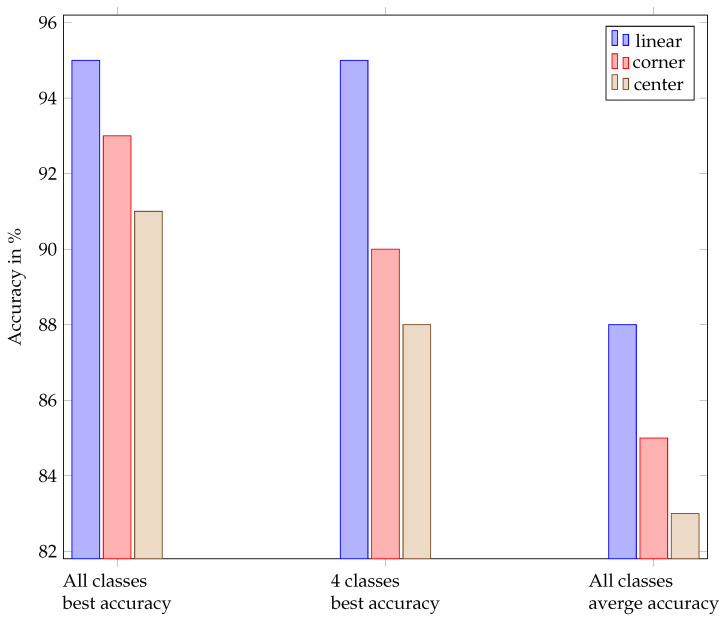
Best accuracy of the best models on all classes compared to the accuracy of the best models on four classes and the average accuracy of all models on each of the three datasets.

The best accuracy average throughout the models was achieved on the linear data, followed by the corner data, as shown in Figure 14.

**Figure 15:**
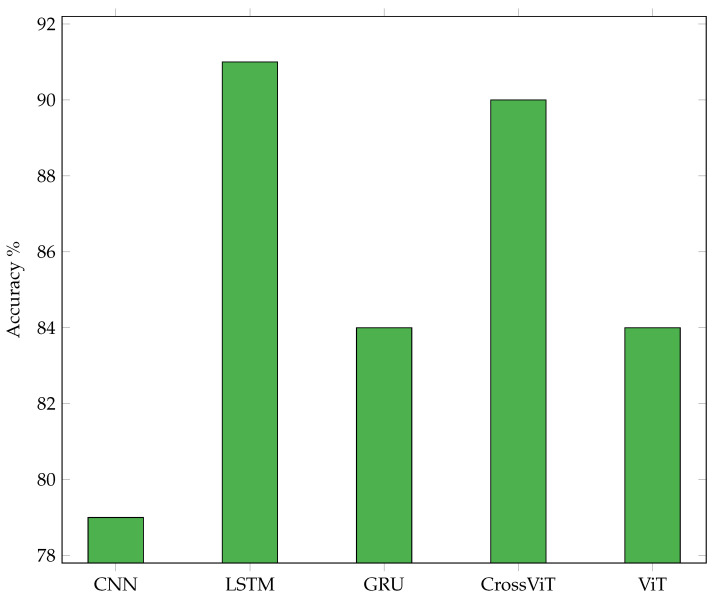
Accuracies per model averaged over datasets.

Results for the evaluation of the averages throughout all datasets are displayed in Figure 15. LSTM showed the highest accuracies. But CrossViT presented comparable accuracies of only 1% less. GRU and ViT performed similarly on average but were 7% less accurate than LSTM. The average performance of CNN was 13% lower than of LSTM.

Time-related information is preserved in the embeddings we utilize for LSTM and GRU by keeping the order of the input. As the input consists of time sequences, the order represents the temporal placement. The embeddings we use are inspired by the embeddings of ViT and CrossViT, which are developed for sequence processing and, therefore, contain the classification token to represent the previous calculations and a position embedding. CNN does not contain a specific token for classification but is being fed the entire gesture at once. It therefore obtains all the data regarding the locations of every pulse train and the echos inside the pulse trains. That means our input is a 1D vector with a size of 768,000 per receiving transducer data per example considered. The best kernel size for this input was found to be 80. Furthermore, we found that average pooling outperforms max pooling. CNN was implemented with a kernel size of four and a stride of two after each convolution layer. The LSTM model yielded the best overall accuracy of 95%, but CrossViT proved almost as good, with an accuracy difference of only 2%. Even though ViT’s model structure is much simpler than CrossViT’s, it nonetheless performed just 4% less accurately than CrossViT. It needs to be mentioned that training LSTM and GRU takes about twice the time compared to training the transformer structures. The number of parameters needed for amplitude-based models can be even lower than the number required for models that use spectrogram images as input, as depicted in Figure 13.

The parameters evaluated for ViT and CrossViT were the number of parallel attention heads, the number of encoder sequence repetitions, the patch size, the dimension of the embedding vector, and the inner-layer dimensionality. The final ViT and CrossViT models for linear and corner data have twelve parallel attention heads, and the one for center data has sixteen. The encoder sequence is repeated 8 times for the linear data and 10 times for the center and corner data. The dimension of the embedding vector of ViT for all datasets is 768. The patch size is chosen to be an integer multiple of the length of one pulse train. In all cases, AdamW optimization, as first introduced by [46], and label smoothing are beneficial. It must be mentioned that the center data have the lowest overall accuracy. The parameters s- and l-branch size only apply to CrossViT due to the more complicated model structure. CrossViT uses three stacks for the s-branch and three stacks for the l-branch in our models throughout its parallel training processes. The number of encoders for the l-branch is decided by conducting multiple trials, but the number of encoders in each stack for the s-branch is fixed at one. The best dimension of the embedding vector in the l-branch is 768 for all datasets. Layer normalization proved to be the most effective technique to improve generalization and to avoid overfitting. AdamW optimization yields better results than Adam optimization. For every dataset, different patch sizes, dimensions of the embedding vector in the s-branch, number of repetitions of the encoding sequence, and number of parallel attention heads show the best results. The number of parameters in CrossViT and ViT are quite similar for the linear and corner dataset. The number for the former is only significantly larger for the linear dataset. But the result of the best CrossViT is much better than the result of the best ViT in the case of the center dataset. For the same number of encoders, CrossViT has better performance than ViT. The average accuracy of CrossViT is very close to that of LSTM, but the number of parameters needed is more than double compared to CNN and ViT. A comparison of the number of needed parameters for classification on the all datasets can be found in the diagram in Figure 13. The dimension of the embeddings for GRU and LSTM was 768. Other parameters under evaluation for the two models were the number of features in the hidden state, the patch size, and the two different embeddings, linear and convolutional, which are described in Section 4. Furthermore, the effect of running the models bidirectionally, label smoothing, and different optimization techniques were evaluated. Independent of the dataset, the best models have 2304 features in the hidden state. For LSTM, AdamW optimization improved the results compared to Adam, and label smoothing was beneficial for all datasets. In combination with the linear dataset, the LSTM model shows the overall best results. With a very modest advantage over CrossViT, this model has the best average accuracy across all datasets. GRU is not as heavy but showed 9% less average accuracy. The best models for GRU, as for LSTM, have different patch sizes for each of the datasets, with no correlation between patch sizes for LSTM and GRU. Convolutional embedding yields the best results for GRU throughout the datasets. When the Adam optimizer is employed without label smoothing, GRU models yield a better result for the center and corner datasets. Compared to LSTM, the GRU model shows noticeably lower accuracy. The effort spent training all of the models for all of the datasets was roughly the same. A comparison of the three evaluated arrangements, depicted in Figure 4 and Figure 5, and classification results, shown in Figure 12, shows that classification of the linear data yields the best results. For all models except CNN, the corner arrangement provides noticeably better classification results in comparison to the center data according to an analysis of the accuracy obtained on both datasets. The diagram in Figure 12 reveals that the center shield never shows the best results. Moreover, Table 8, Table 9, Table 10, Table 11 and Table 12 show that classification of tap versus swipe gestures permits significantly better results than differentiation of swipes.

## 6. Discussion

Our suggested reason why the first few gesture frames of each gesture per person are more difficult to classify is that the user may take some time to become familiar with the gestures at first. Therefore, we assume that those gesture frames have more variability.

Clearly increased accuracy was reached for classifications of swipes versus taps. That means the characteristics of the echo data of swipes contains meaningful differences compared to the echo data of taps. Tap gestures have higher changes in frequency per time and come with higher echo amplitudes as the hand is at about the center of the device, and the changes of the echo size throughout pulse trains are more noticeable compared to swipes. The other classification that led to a similarly high accuracy was found for the classification of the swipe angle: (sl, sr) versus (su, sd). Therefore, we conclude that the differences in the characteristics of the echo data between angles of a straight movement are more obvious than the disparities amid straight movements at the same angle in the opposite direction. As the accuracy of the four gestures, pp, dt, su, and sd, depicted in Figure 6, compared to the accuracy of the six gestures, pp, dt, su, sd, sr, and sl, for the center and corner data shows, there is no improvement if only four gestures are evaluated. That goes in line with our findings that it is more difficult to distinguish the direction of a swipe or to count the number of taps compared to determining the angle of the movement axis. If (pp, dt) vs. (sd, su) vs. (sr, sl) are chosen as three classes, an accuracy of more than 90% can easily be achieved. Comparing different models on average over all datasets, LSTM and CrossViT reached the best accuracies. But those are, at the same time, the models that require the highest number of parameters. Training times for LSTM and GRU are significantly higher than for CrossViT and ViT. As the best accuracy of CrossViT versus LSTM models only varies by 1%, CrossViT seems preferable in most cases: not to mention that the accuracy reached on the center and corner data are better than on linear data. With GRU and ViT, 9% lower accuracy was obtained. Among those two models, GRU requires substantially more training time. Furthermore, the ViT models demand a lower number of parameters. For this reason, ViT is preferable in most cases. As GRU and ViT require fewer parameters, whenever the size of the model has to be taken into consideration, i.e., for embedded and edge applications, ViT seems the best choice, but LSTM is preferable if the size of the model is not crucial. Assuming that the model size is very critical, CNN models, with the lowest parameter requirements amidst all trained models, can be taken into account. Specifically, for the linear dataset, CNN shows a decent accuracy of 83%. The best results for CrossViT and ViT were acquired with 12 parallel attention heads for linear and corner data and 15 for center data, which might imply that for noisier training data, a higher number of attention heads is beneficial. As only the best model for linear data relies on bidirectional training, we cannot conclude that it has a significant positive effect. The classification of corner data compared to center data was more successful. We infer that a straight line between the sender and receiver in the orientation of the swipe is beneficial, and that higher distances between the sender and receiver give the echo data better differentiable characteristics. Therefore, a corner arrangement is preferable to a center arrangement for classification of the hand gestures regarded in our setup. The most significant differences between the CNN models are the types of normalization sub-layer and pooling sub-layer and, generally, the number of layers. The reason why the CNN model showed the worst accuracy compared to all others might be because this type of structure does not come with an internal memory. Therefore, the time-dependent information of the gestures is not well learned for CNN structures, and feeding the entire gesture at once is not sufficient to allow the model to perform as well as models with a memory, which are designed for sequential data. While LSTM and GRU cope with the vanishing and exploding gradient problem, these models need a lot of training time, which increases significantly with each added layer. ViT and CrossViT take significantly less time, have half of the number of trainable parameters, and achieve almost the same accuracy. Therefore, these structures are probably preferable in most cases. The comparison of models with spectrogram images as input and the models without the Fourier transform with respect to model size indicates that, at least for now, doing the Fourier transform as pre-processing results in a much smaller model. Therefore, it might be advisable in most cases to do this transformation before classification. The Fourier transform accentuates frequency changes, making it possible to assess the Doppler effect. Since some information is inevitably lost when calculating the spectrogram, it might be possible that, for some other gestures, the information given by a spectrogram is not sufficient. The superior classification results obtained on the linear dataset might be due to the fact that only four gestures are being classified in this case. Furthermore, the distance between the sending transducer and the farthest receiving transducer is highest in this arrangement. That might allow for more accurate classification of the swipe gestures. Additionally, the data that are evaluated contain more redundancy, as the two evaluated transducers should show roughly the same changes to echo length, height, and position since they are linearly placed. On the other shields, only one of the transducers is in linear alignment with respect to the swipe gestures. Thus, despite using more information for classification for the center and the corner data, classification of the linear data yields the best results. The corner data allow for significantly better classification accuracies for all but the CNN model compared to the center data. This might mean that linear placement of the transducers with respect to the classified gestures is beneficial and supports the suggestion that a higher distance between the sending transducer with respect to the receiving transducers is favorable.

## 7. Conclusions

Five different ML model types were trained on three different datasets for four or six gestures depending on the shield arrangement. All datasets were acquired with dedicated HW comprising four ultrasonic transducers in different arrangements. The main novelty presented is that the data exclusively contain raw ultrasound echo data. That means the processing is purely ML-based, and the processing-intensive Fourier transform is left out. Features are learned by the algorithms autonomously. The ML models tested include convolution-, sequential-, and attention-based models. For all models, dedicated adjustments were successfully implemented to feed them raw ultrasound amplitudes. For LSTM and GRU, ViT- and CrossViT-inspired embeddings were implemented to allow autonomous feature extraction. For the same goal using CNN, a suitable kernel size was found. The experiments outlined in Section 4 and carried out with the HW described in Section 3.1 allow a comparison of the different arrangements of the transducers on the one hand and a comparison of the distinct ML models on the other hand with respect to the various gestures. It was shown that adjustment of the models to the specific new task of evaluating raw ultrasound data was successful. The gestures consisted of taps perpendicular to the transducers and two types of swipes horizontal to the transducers. Depending on the arrangement, either two or four types of swipes were classified. It was found that sequential and attention-based models are superior to convolution-based models for ultrasonic HGR. This confirms that temporal changes are very important for HGR. Regarding the transducer arrangement, corner and linear were superior to center, which leads to the inference that a higher distance between the sending and the receiving transducer is beneficial for ultrasound HGR. This goes in line with the expectation that the data received by a transducer that is closer to the transmitter are distinct from the data received by a transducer that has a higher distance from the transmitter. These differences are increased with larger differences in distance from the transmitter. Due to its symmetry, the center arrangement is therefore less suitable for an HGR system. That LSTM outperforms GRU and CrossViT surpasses Vit signifies that ultrasound HGR on raw input data is a complex task that, up to now, has demanded a complex ML model. The maximum accuracy achieved in this work is 95% for four gestures, 93% for six gestures, and up to 100% accuracy on differentiating tap vs. swipe gestures. This accuracy is similar to the results reported by current research. However, it must be noted that the complexity of ultrasound-based HGR depends highly on the type, amount, and arrangement of sensors of the HW used as well as the type of gestures that are being evaluated. As Table 2 highlights, HGR systems based on sensor fusion, vision, and radar are able to differentiate higher numbers of more fine-grained gestures with similar accuracy. Similar to the proposed research, [13,14] use time-efficient processing with DL on raw data in the time domain for HGR. Classification by [26,28] relies on the Doppler effect and is therefore constrained to tap movement recognition. Papers [29,30,33] require the Fourier transform and more complicated signal processing. Paper [24] requires eight transducers, processing intensive beamforming, and oscilloscopes for actuation and read-out. The authors of [25] develop a system that reaches higher resolution and distance but is based on a 64-element array of bigger transducers. Finally, [23] requires the user to wear a device. The authors of [32] achieve 95% to 100% accuracy, but their method requires an array of seven transducers placed in a circular arrangement. Therefore, the evaluation of Doppler shifts is sufficient for the evaluation of the direction of arrival of a hand. Up to now, ultrasound-based HGR is in the range of 86% to 100%, as [28] with 86%-100%, [29] with 97%, and [30] with 97% show. Our approach shows that similarly high accuracy can be achieved without using the Fourier transform, as Table 3 underlines. Raw ultrasound data are significantly different from video, radar, WiFi, or other available hand gesture data. As a consequence, our research relies on significantly different processing. The dataset used for training contains low office noise and noise from common machines like a laptop, screens, and similar devices. Different hand shapes, gesture speeds, direction of the palm up and down are considered, as different persons took part in the data acquisition. Nevertheless, it is required that the palm be in a parallel position to the array to generate a significantly high echo. Further, the gesture must be carried out in a range of about 15 to 40 cm distance. In our case, no object obstructed the FOV. But as the FOV is comparatively small, this is not expected to pose a significant problem in real-world use. Possible applications of the gesture recognition algorithms are smart speakers or automotive interiors. While radar applications face the problem of many phantom objects, the range and FOV of our ultrasound application are small enough to avoid these problems. Already installed speakers and microphones could potentially be enhanced by the proposed application. It has to be considered that we only evaluated data captured by two or three ultrasound transducers, which do not contain sufficient information to distinguish fingers let alone differentiate finger positions. For such tasks, more transducers and a stronger emitter, as in [25], are required. Yet the scope of this work was to show that HGR based on ultrasound data is possible in the time domain only and with very small HW requirements. The presented results demonstrate that it is possible to do classification of ultrasound-based gestures without Fourier transform by utilizing a wide variety of deep learning models when the models and incoming data are adjusted adequately, as described in Section 4.

As a next step, we want to explore new models like spiking neural networks. It needs to be confirmed if a combination of spiking neural networks with Fourier-transform-free classification is possible. Such a concept would be particularly advantageous for implementations on devices with low processing power. Considering that the output size is quadratically dependent on the input size, shortening the input sequences would likely result in a large reduction in the size of the transformer network. Retaining more information related to time seems to be a field that needs more research, as it might benefit the determination of the direction of swipes.

Additionally, it might be possible to apply the presented approach to do classification after every pulse train, which would permit earlier gesture prediction. 

## Figures and Tables

**Table 1:** Challenges of ultrasound HGR systems.

Type	Reason	Challenge
Physics of airborne ultrasound	Wind, humidity, temperature	Different speeds of ultrasound waves
Physics of airborne ultrasound	Ultrasound waves are mechanical waves transmitted and received by a membrane	Echo is not a single peak
Physics of airborne ultrasound	High attenuation of ultrasound in air	Low SNR
Physics of airborne ultrasound	Surrounding static objects	Static echos
Physics of airborne ultrasound	Repeated reflection	Phantom echos
HW specific	Low number of transducers	Low resolution
HW specific	Low echo delay differences between receivers	Low distance between receivers
HW specific	Low emitting power of the sender	Low SNR
HW specific	Transducer instead of separate sender and receiver	Lower sending power, lower receiver resolution, lower SNR
HW specific	Transceiver noise	Low SNR
General	No public training data	No pre-training of model on public dataset

**Table 2:** SotA comparison of the considered sensor types for HGR.

Sensor Type	Pre-Processing	Classification	Accuracy	Number of Gestures	Type of Gestures	Reference
Vision	CNN based, skin color detection, morphology, background subtraction, raw image data, color space, Gaussian mixture model, semantic-segmentation-based deconvolution NN, Canny operator edge detection, transfer learning, color cloud, neural gas, directional active model	CNN, GRU, LSTM, symmetric-positive-definite-manifold-based NN, softmax-classifier, SVM, transfer learning, SNN	85.3–100%	7 to 14	Custom hand gestures, hand tracking, hand edge detection	[5,6,7,8,9,10,11]
Radar (pulsed, Doppler, FMCW, other actuation)	Time-Doppler, range-Doppler (most common), time-velocity, range-amplitude, time-range, time-amplitude, time-RCS, point cloud	CNN, energy estimation, HMM, RNN, random forest, naive Bayes, kernel estimators, SVM, QEA, kNN, conditional statements, k-means clustering, GoogLeNet, observing back-scattered waves, SNN	82 to 99.5%	2 to 15	Letters from sign language, finger counting, digit writing, static hand postures, midair signature tracking, custom hand gestures,	[12,13,14]
WiFi (RSSI, CSI, FMCW)	Signal strength, channel conditions, properties of wireless links, frequency shift, angle of arrival	CNN, DTW, SVM, self-attention-based	51 to 99.69%	4 to 11	Finger, hand, arm gestures, hand tracking, fall detection	[15,17,18,19,20,21,22]
Ultrasound	Raw amplitude values	CNN, GRU, LSTM, ViT, CrossViT	85% to 95% (100% for tap vs. swipe)	4 to 6	Hand gestures	This paper

**Table 3:** SotA comparison of approaches using ultrasound for HGR; simple gesture = movement in one direction, macro = whole hand involved; complex = at least two directions in one gesture; micro = finger gestures.

Type of Actuation	Pre-Processing	Classification	Accuracy	Number of Gestures	Type of Gestures	Reference
pulsed single frequency	TOF	MLP, LSTM, CNN	92.87%	7	simple, macro	[26]
continuous, single-frequency wave	spectrogram	thresholds	94.7% (home), 94.3% (cafe)	8	simple, macro	[28]
pre-defined training sequence	spectrogram and CIR	CNN	97.92%	12	complex, fine	[29]
pulsed single frequency	spectrogram	CNN	96.7%/97.8%/95.5%	8/4/4	all /macro, simple/micro, simple	[30]
pulsed single frequency	spectrogram, dynamic speed warping pattern matching	one-shot learning, kNN,	up to 99.36% (four users for training)	9	simple and complicated, macro	[31]
continuous single frequency	spectrogram, delay-and-sum beamforming	temporal convolutional network (TCN)	93% to 100%	6	simple, macro	[32]
pulsed single frequency	spectrogram, TOF	CNN	91% to 93%	4	complex, macro	[33]
pulsed frequency bursts	inter-node distance calculation	HMM	83.8%	7	complex, macro	[23]
pulsed single frequency	raw amplitude values	LSTM, GRU, CNN, CrossViT, ViT	85% to 95% (100% for tap vs. swipe)	4 to 6	complex, macro	this paper

**Table 4:** Advantages and drawbacks of the considered sensor types.

Sensor Type	Advantages	Drawbacks	Frequency	Power Consumption	System Size
Vision	Many already available image processing tools	Privacy concerns, sensitive to low light and fog	400 THz to 700 THz	Microsoft Kinect V2: 15 W, Intel RealSense SR300: 1.8 W	Microsoft Kinect V2: 66 × 249 × 67 mm, Intel RealSense SR300: 110 × 12.5 × 3.75 mm
Radar	No privacy concerns, better accuracy than ultrasound and WiFi	Higher power consumption than ultrasound, lower resolution than high-frequency ultrasound applications, sensitive to electromagnetic interference, too cost-intensive for the target market, design bigger than ultrasound	5 MHz to 130 GHz	Novelda X4: 120 mV, Infineon BGT23MTR12: 660 mW	Novelda X4: 3.283 × 2.627 mm, Infineon BGT23MTR12: 5.5 × 4.5 mm
WiFi	HW available in most indoor environments	Simultaneous use of WiFi for communication and localization difficult, changes in environment cause a need for retraining	2.4 GHz, 5 GHz, and 6 GHz	Archer AX55: 4.8 W	Archer AX55: 261 × 135 × 41 mm
Ultrasound	Economical, luminance-invariant, no electromagnetic interference, power-saving, extremely compact design, slower propagation speed	Lower accuracy than radar for low frequencies (as used in this paper) and vision-based systems, higher attenuation in air, simultaneous use for audio and ultrasound difficult, less-investigated	20 kHz to 18 MHz	PCB sizes: linear: 120 × 40 mm, center, corner: 70 × 70 mm, sensor size: 2.5 × 3.6 × 1.0 mm	9 mW (four transducers)

**Table 5:** System parameters.

Parameter	Value
Sent frequency	24 kHz
Pulse repetition time	16 ms
Pulse length	5 wavelength
Number of pulse trains per gesture frame	250
Sampling frequency	192 kHz
Number of transducers in transmit mode	1
Number of transducers in receive mode	3

**Table 6:** Overview of datasets and models used for classification.

Property of Data Set	Value of Property of Each Data Set
Number of gestures	Linear: 4
	Center, corner: 6
Gestures	Linear: push pull, double tap, swipe up, swipe down, as depicted in Figure 6
	Center, corner: same as linear, additional gestures: swipe right, swipe left, also as depicted in Figure 6
Number of gesture frames per dataset	Linear: 899
	Center: 898
	Corner: 900
Number of participants	Linear: 9
per dataset	Center, corner: 6
Number of pulse trains per gesture	All datasets: 250
Training and validation split	Manually for more reliable validation; see reasoning in Section 5
Tested model types	CNN, LSTM, GRU, ViT, and CrossViT

**Table 7:** Comparison of highest accuracy per model.

Model Name	Accuracy in %
CNN	83
GRU	86
LSTM	95
ViT	89
CrossViT	93

**Table 8:** Confusion matrix of the best accuracy of GRU on the linear shield data. All values are expressed in %.

	dt	pp	sd	su
**dt**	96	4	0	0
**pp**	0	98	2	0
**sd**	0	0	86	0
**su**	0	0	21	79
Average accuracy				90
Tap vs. swipe				100

**Table 9:** Confusion matrix of the best accuracy of LSTM on the linear shield data. All values are expressed in %.

	dt	pp	sd	su
**dt**	96	4	0	0
**pp**	7	93	0	0
**sd**	0	0	94	5
**su**	0	0	4	96
Average accuracy				95
Tap vs. swipe				100

**Table 10:** Confusion matrix of the best accuracy of CNN on the linear shield data. All values are expressed in %.

	dt	pp	sd	su
**dt**	92	6	2	0
**pp**	16	83	0	0
**sd**	0	2	86	12
**su**	1	0	20	77
Average accuracy				85
Tap vs. swipe				98

**Table 11:** Confusion matrix of the best accuracy of ViT on the corner shield data. All values are expressed in %.

	dt	pp	sd	su	sl	sr
**dt**	89	11	0	0	0	0
**pp**	14	83	3	0	0	0
**sd**	3	6	81	11	0	0
**su**	3	3	3	89	0	3
**sl**	0	0	0	0	97	3
**sr**	0	0	0	0	6	94
Average accuracy						89
Tap vs. swipe						98
sd, su vs. sl, sr						96

**Table 12:** Confusion matrix of the best accuracy of CrossViT on the corner shield data. All values are expressed in %.

	dt	pp	sd	su	sl	sr
**dt**	92	3	6	0	0	0
**pp**	9	78	14	0	0	0
**su**	0	3	97	0	0	0
**sd**	0	3	3	94	0	0
**sl**	0	0	0	0	100	0
**sr**	0	0	0	6	0	94
Average accuracy						93
Tap vs. swipe						95
sd, su vs. sl, sr						97

## Data Availability

Data are contained within the article.
